# Shrub Invasion Decreases Diversity and Alters Community Stability in Northern Chihuahuan Desert Plant Communities

**DOI:** 10.1371/journal.pone.0002332

**Published:** 2008-06-04

**Authors:** Selene Báez, Scott L. Collins

**Affiliations:** Department of Biology, University of New Mexico, Albuquerque, New Mexico, United States of America; University of Zurich, Switzerland

## Abstract

**Background:**

Global climate change is rapidly altering species range distributions and interactions within communities. As ranges expand, invading species change interactions in communities which may reduce stability, a mechanism known to affect biodiversity. In aridland ecosystems worldwide, the range of native shrubs is expanding as they invade and replace native grassland vegetation with significant consequences for biodiversity and ecosystem functioning.

**Methodology:**

We used two long-term data sets to determine the effects of shrub encroachment by *Larrea tridentata* on subdominant community composition and stability in formerly native perennial grassland dominated by *Bouteloua eriopoda* in New Mexico, USA.

**Principal Findings:**

Our results indicated that *Larrea* invasion decreased species richness during the last 100 years. We also found that over shorter temporal scales species-poor subdominant communities in areas invaded by *Larrea* were less stable (more variable in time) compared to species rich communities in grass-dominated vegetation. Compositional stability increased as cover of *Bouteloua* increased and decreased as cover of *Larrea* increased.

**Significance:**

Changes in community stability due to altered interspecific interactions may be one mechanism by which biodiversity declines in grasslands following shrub invasion. As global warming increases, shrub encroachment into native grasslands worldwide will continue to alter species interactions and community stability both of which may lead to a decline in biodiversity.

## Introduction

The measurement of ecological stability and the relationship between community stability and diversity have been the subject of much recent theoretical debate and empirical analysis [1]. In the past, empirical studies of stability were hindered by a lack of long-term data, yet such datasets are beginning to accumulate in both experimentally- and naturally-assembled communities. Both theory and manipulative experiments have demonstrated a positive relationship between community stability and species diversity in ecological communities [1]–[3]. Critical evidence for this relationship occurs primarily in competitive communities (e.g., producers) where species diversity and the structure of species interactions affect species composition and turnover [4], [5], population stability (e.g., rates of variation in population densities) [6], [7], and community stability (e.g., temporal variation in net primary production) [2], [8]. However, empirical research exploring how specific changes in the structure of species interactions alters multiple aspects of community stability, including species diversity and the temporal rates of population and community change, are still needed.

Theoretical and empirical research have shown that species invasions and extinctions in competitive communities can affect community compositional stability (e.g., temporal variability in species diversity) because they can lead to further events of species extinction or invasion [1], [9], [10]. Mathematical models attribute these changes in community stability to shifts in the structure of species interactions, with larger structural shifts leading to lower community compositional stability [2], [10]–[15]. Therefore, the mode in which an invading species interacts is expected to be a crucial factor affecting species diversity and community compositional stability in competitive communities [1], [3], [14], [16]–[18]. Field studies assessing the effects of species invasion on various aspect of community stability are needed to validate predictions based on theoretical models and to identify community and population parameters that affect community compositional stability.

In arid and semiarid regions species interactions are important factors structuring the diversity of plant communities [19]–[22]. Both competition and facilitation imposed by dominant species affect temporal variation in the cover and species composition of subdominant plant communities [6], [23], [24]. Therefore, in aridland ecosystems, invasion or extinction of facilitating or competing dominant plant species may have a particularly strong effect on various measures of the temporal stability of subdominant plant species. Community instability or compositional community instability can be defined as the gain or loss of species or changes in species abundances that result in large directional changes in community composition and diversity [25]. Temporal variability in population and community parameters, including rates of change in population density and rates of species turnover, also may occur following species invasions. Thus, population and community instabilities linked to invasion and extinction events can yield valuable insights into the mechanisms responsible for maintaining species diversity and certain aspects of community stability in time [1], [2], [4], [26].

During the last century, C_4_-dominated grasslands worldwide have experienced dramatic and rapid ecological changes due to encroachment by native C_3_ shrubs [27]–[29]. In southwestern North America more than 19 million hectares of arid and semi-arid C_4_-dominated grassland have been invaded by *Larrea tridentata* (creosote bush), a common C_3_ shrub native to North American desert ecosystems [28]. Shrub invasion promotes loss of biodiversity and impacts various aspects of ecosystem functioning [28]–[33]. *Larrea* invasion is thought to result from multiple drivers including overgrazing, altered disturbance regimes, elevated atmospheric CO_2_, and especially altered precipitation regimes and increased temperatures [28], [34]. Indeed, the northern distribution of *Larrea* is known to be limited, in part, by winter night time low temperatures which have increased by as much as 2°C over the last century in parts of the US southwest [35], [36]. Because climate change is potentially an important driver of shrub encroachment, this phenomenon is likely to continue across arid and semi-arid grasslands worldwide.

Here, we explore the effects of *Larrea* invasion on the local extinction of the native C_4_ grass *Bouteloua eriopoda*, and the population and community stability of subdominant species in a northern Chihuahuan Desert ecosystem. We hypothesize that changes in species interactions due to the replacement of the competitive grass *Bouteloua*, with the predominantly facilitative shrub *Larrea* [19], [37], [38] result in altered patterns of population and community stability. To test this hypothesis we used long-term (1995–2004) vegetation data from permanent plots to quantify population, compositional, and community cover stability [2], [39] in pre-invasion *Bouteloua*-dominated plant communities and post-invasion *Larrea*-dominated communities at the Sevilleta National Wildlife Refuge (SNWR), central New Mexico, USA. We also used data from 1915 through 2001 to examine the effects of *Larrea* invasion on species diversity in *Bouteloua*-dominated communities at the Jornada LTER in southern New Mexico, USA. Understanding the effects of invasion on community stability is particularly important given the current global trends of biodiversity loss and changes in species distribution and abundance [4].

## Methods

We used two long-term datasets to evaluate the effects of shrub invasion on richness and dynamics of subordinate species in Chihuahuan desert plant communities. Historical photographs from the SNWR show that *Larrea* has invaded formerly C_4_ dominated grasslands during the last 100 years, primarily displacing the native C_4_ grass *Bouteloua eriopoda* (black grama). In this area, vegetation invaded by *Larrea* has consistently 20–30% fewer species of vascular plants per m^2^ than adjacent non-invaded grasslands dominated by *Bouteloua* (14.05 SE 0.52 vs. 21.4 SE 0.59 1-m^2^, respectively; Repeated Measures MANOVA, *F*_1,14_ = 26.27, *P*<0.001; [40]). Thus, to test for differences in community and population stability following shrub invasion, we used 10-years (1995–2004) of vegetation measurements in permanent plots located in native grassland and nearby areas currently dominated by *Larrea* but formerly dominated by C_4_ grasses at the SNWR. Our study sites were located 4 km apart, with the permanent vegetation plots in grass vegetation located 1 km north of the current grass-shrub transition zone, and the shrub invaded plots were 3 km south of the transition zone. The SNWR is a 100,000 ha area located along the Rio Grande in Central New Mexico. Mean annual temperature at the study site (1989–2006) is 13.2°C and the average annual precipitation is 255 mm, of which 60% occurs during the monsoon season from July through September. In addition, to assess the effects of *Larrea* invasion on plant species diversity in *Bouteloua*-dominated grassland we used long-term (1915–2001) vegetation measurements taken in permanent quadrats in C_4_-dominated grassland that underwent shrub invasion around 1960 at the Jornada Experimental Range (JER) in south-central New Mexico [41], [42] approximately 270 km south of the SNWR. Mean annual temperature and average annual precipitation at the JER are similar to values at the SNWR [41].

For the community stability analyses we used annual data from 1995 to 2004 collected in 193 permanently located 1-m^2^ quadrats in a native grassland community dominated by *Bouteloua eriopoda* (black grama), and in a former grassland area (prior to 1950) that is now dominated by *Larrea tridentata* (creosote bush). At the grassland and shrubland sites, quadrats were located in four replicate 36×36 m plots. In each plot vegetation was measured in 36 permanently located 1-m^2^ quadrats placed five meters apart in an evenly spaced 6×6 grid. The cover of all plant species was measured annually using a 1-m^2^ frame divided into 10 cm units to facilitate cover estimates (for further details on the experimental layout see [40]). Within each site we restricted our analyses to plots where the dominants, *Larrea* or *Bouteloua*, had a minimum mean cover of 0.1% over 10 years. A total of 193 1-m^2^ quadrats met this criterion, 100 of which were located in *Bouteloua*-dominated grassland, and 93 in *Larrea*-invaded shrubland. In this system, as in other aridland ecosystems, grass- and shrub-dominated areas resulted in spatially structured communities in which soil moisture and nutrients are concentrated in “islands of fertility” beneath patches of vegetation [43]–[45]. In both vegetation types annual forbs and grasses are common. Shrub-dominated vegetation, in particular, has a relatively high cover of large seeded winter annual forbs. In contrast, grass-dominated vegetation has high richness and cover of small-seeded annual forbs, which are primarily present during summer [46]. The annual cover of all plant species in each quadrat was estimated in May when winter annuals peak in abundance and biomass, and again in September when perennial and summer annual species peak in abundance and biomass in response to the summer monsoon. Cover for each species was expressed as a percentage of the summed total of species maximum cover values in each quadrat in each year.

In addition, we used data from the JER to determine if plant species diversity declines as *Larrea* invasion occurs or if *Larrea* invades areas that are already low in species diversity. To do so, we evaluated changes in species diversity in nine permanently located 1-m^2^ quadrats where species composition was recorded at irregular time intervals between 1915 and 2001 [41]. All quadrats were originally dominated by *Bouteloua eriopoda* in 1915 but are now completely dominated by *Larrea tridentata*.

### Data analyses

All analyses of data from the SNWR were conducted by pooling quadrats across the four blocks within each vegetation type and considering the quadrats as independent samples [47]. Pooling samples was deemed appropriate because cover of dominant species was randomly distributed within plots and blocks, quadrats were separated by distances of 5 to 272 m (i.e., 5 to 272 times the area of a sampling unit), and there were no significant block effects on *Larrea* (R^2^ = 0.002, *F*_3,96_ = 0.066, *P* = 0.97) or *Bouteloua* (R^2^ = 0.022, *F*_3,88_ = 0.073, *P* = 0.54) cover.

Ecological stability has many different definitions and metrics [1], [25]. In this study, we quantified four measures of ecological stability: cover stability, compositional stability, population stability, and species turnover as defined below. Following Tilman et al. (2006), *cover stability* of the subdominant community was measured as the mean cover divided by the temporal standard deviation of cover. *Compositional stability* was measured as the mean of the Euclidian distances calculated for all the possible temporal pair comparisons of the subdominant community in each quadrat (i.e., yr 1 vs. yr 2, yr 1 vs. yr 3, yr 1 vs. yr 4… and yr 9 vs. yr 10 [25]) and multiplied by −1 to make the variable more intuitive in terms of stability. Because indices of stability may be affected by differences in species richness, we used the log-transformed residuals of a linear regression of log-transformed compositional stability vs. mean species richness in our ANCOVA models to eliminate possible effects of species richness on this measure of stability. *Turnover* was the sum of the probabilities of colonization and extinction for each species that occurred in a quadrat. Turnover was calculated using a Markovian chain probabilistic function (colonization λ+extinction δ, where λ = *k*/*k*+*l* and δ = *m*/*m*+*n*, where *k* number of colonization events, *l* = number of stage persistence events from absent to absent, *m* = number of extinction events, *n* = number of presence persistence events [48]. In addition, we evaluated the *population stability* of subdominants by dividing the mean cover of each species over 10 years by the temporal standard deviation of cover for that species and taking the mean of these values [2].

Analysis of covariance (ANCOVA) and linear regressions were used to assess the effects of the dominant species, *Bouteloua* and *Larrea*, on subdominant population and community stability at the quadrat scale. ANCOVA models were used to evaluate the dynamics, structure, and species richness of subdominant communities as a function of vegetation type (i.e., site), cover of the dominant species, *Bouteloua* or *Larrea*, and the interaction between site and cover of dominants. The temporal mean cover of *Bouteloua* and *Larrea* was used to evaluate the effect of the dominant species on the subdominant communities of a given quadrat because cover fluctuated from one year to the next but there was no overall net change in cover of the dominant species over the study period [40]. We used ANCOVA analyses to evaluate whether *Bouteloua* and *Larrea* had similar effects on subdominant community structure and stability. Thus, we assessed how the cover of the dominant species affected the cover and species richness (10-year mean of species present in a given quadrat) of subdominants as a whole, as well as by life form (forbs, grasses and shrubs), life history, (summer annuals, winter annuals, and perennials), and distribution (shared between grass and shrub vegetation, or restricted to either vegetation type). Most of the dependent variables were square root or log-transformed to achieve normality.

## Results

**Figure 1:**
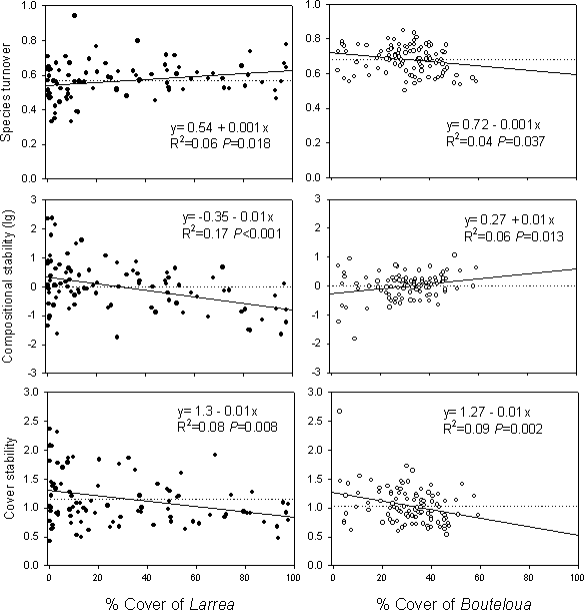
Linear regressions (solid lines) and means (dotted lines) of stability and species turnover of subdominant communities as a function of the cover of *Larrea* and *Bouteloua*, at the Sevilleta National Wildlife Refuge, New Mexico, USA.

At the SNWR, fifty-six percent of subdominant species were found in both grass- and shrub-dominated vegetation (Jaccard index of dissimilarity = 0.44). In each community, the cover of the dominant species affected the stability, structure, and species richness of the subdominant communities (Table 1, Figure 1). A significant site by dominant species cover interaction indicated that *Bouteloua* and *Larrea* had opposite effects on the directionality of compositional stability (Table 1, Figure 1). As cover of *Bouteloua* increased, compositional stability of subordinate species increased due to decreased turnover rates. In contrast, as cover of *Larrea* increased, compositional stability of subordinate species decreased and turnover rates increased. However, both *Bouteloua* and *Larrea* had a negative effect on cover stability of the subdominant communities as a whole (*F*_1,99_ = 5.7, *P* = 0.02, Tables 1, 2). That is, variation in the total cover of subdominant species increased as cover of the dominants increased in both grass- and shrub-dominated vegetation. In addition, high cover values of *Bouteloua* significantly decreased the population stability of subdominant species (i.e., variations in the cover of subdominant species increased), whereas the cover of *Larrea* had no effect on population stability of subdominants (Tables 1, 2).

**Table 1:** ANCOVA results for the effects of vegetation type (site) and cover of *Larrea* and *Bouteloua* on the population and community stability of subdominants.

Factors	Estimates						Whole model	
	Term	d.f.	Estimate	SE	t -ratio	*P*	r^2^	*P*
**Population stability**	Intercept		0.454	0.009	50.29	<0.001	0.06	0.012
	site	1	−0.006	0.004	−1.4	0.163		
	cover	1	−0.0004	0.0002	−1.83	0.068		
	site x cover	1	0.001	0.0002	3.03	0.002		
**Cover stability**	Intercept		1.283	0.061	21.1	<0.001	0.10	<0.001
	site	1	0.059	0.028	2.05	0.041		
	cover	1	−0.006	0.001	−3.49	<0.001		
	site x cover	1	0.001	0.001	0.85	0.394		
**Compositional stability (log)**	Intercept		0.04	0.095	0.42	0.672	0.14	<0.001
	site	1	−0.001	0.045	−0.02	0.986		
	cover	1	−0.001	0.002	−0.53	0.594		
	site x cover	1	−0.01	0.002	−3.7	<0.001		
**Turnover**	Intercept		0.63	0.013	46.05	<0.001	0.32	<0.001
	site	1	−0.056	0.006	−8.76	<0.001		
	cover	1	−0.0002	0.0003	−0.53	0.593		
	site x cover	1	0.001	0.0003	2.74	0.006		
**Colonization**	Intercept		0.331	0.01	36.72	<0.001	0.27	0.004
	site	1	−0.032	0.004	−7.66	<0.001		
	cover	1	−0.0001	0.0002	−0.71	0.48		
	site x cover	1	0.0007	0.0002	2.85	0.004		
**Extinction**	Intercept		0.297	0.007	40.62	<0.001	0.25	<0.001
	site	1	−0.027	0.003	−7.87	<0.001		
	cover	1	−0.0001	0.0002	−0.34	0.735		
	site x cover	1	0.0002	0.0002	1.4	0.163		

Compositional stability was logarithmically (log) transformed.

**Table 2:** Means, Standard Errors, and estimates of linear regression of community stability, structure, and species richness of subdominant species as a function of the cover of the dominant species, *Larrea tridentata* and *Bouteloua eriopoda* over a 10 year period at the Sevilleta NWR.

	*Larrea*					*Bouteloua*				
Stability	Mean (SE)	Equation	r^2^	*P*	n	Mean (SE)	Equation	r^2^	*P*	n
Population stability	0.43(0.01)	y = 0.42+0.0003x	0.02	0.228	92	0.44(0.004)	y = 0.48−0.001x	0.13	<0.001	100
Community stability	1.16(0.05)	y = 1.30−0.01x	0.08	0.008	90	1.04(0.03)	y = 1.27−0.01x	0.09	0.002	100
Compositional stability (log)	3.95^−16^(0.09)	y = 0.35−0.011x	0.17	<0.001	91	1.10^−15^(0.04)	y = −0.27+0.01x	0.06	0.013	100
Probabilites of turnover	0.57(0.01)	y = 0.54+0.001x	0.06	0.018	91	0.68(0.007)	y = 0.72−0.001x	0.04	0.037	100
Probabilities of colonization	0.29(0.01)	y = 0.28+0.001x	0.05	0.03	93	0.36(0.004)	y = 0.39−0.001x	0.07	0.008	100
Probabilities of extinction	0.27(0.01)	y = 0.26+0.0002x	0.01	0.257	93	0.32(0.004)	y = 0.33−0.0004x	0.01	0.243	100
**Structure and richness**										
*Cover according to life forms*										
Forbs (sqrt)	1.1(0.05)	y = 0.85+0.01x	0.25	<0.001	92	1.34(0.04)	y = 1.66−0.01x	0.12	<0.001	100
Grasses (sqrt)	1.58(0.12)	y = 1.55+0.001x	0.001	0.83	75	1.64(0.06)	y = 1.87−0.01x	0.02	0.145	97
Shrubs (sqrt)	1.43(0.095)	y = 1.22+0.01x	0.06	0.058	57	1.15(0.05)	y = 1.55−0.01x	0.09	0.002	95
*Cover according to life history*										
Summer annuals (sqrt)	1.03(0.06)	y = 0.86+0.01x	0.08	0.007	88	1.51(0.04)	y = 1.76−0.01x	0.06	0.018	100
Winter annuals (sqrt)	1.08(0.06)	y = 0.76+0.01x	0.39	<0.001	91	1.03(0.04)	y = 1.19−0.01x	0.03	0.113	99
Perennials (sqrt)	1.5(0.09)	y = 1.40+0.003x	0.01	0.319	83	1.45(0.04)	y = 1.88−0.01x	0.16	<0.001	100
*Cover according to distribution*										
Restricted (log)	0.51(0.07)	y = 0.25+0.01x	0.27	0.002	33	0.34(0.04)	y = 0.47−0.004x	0.03	0.218	52
Shared (log)	0.43(0.03)	y = 0.37+0.002x	0.07	0.01	92	0.5(0.01)	y = 0.65−0.01x	0.22	<0.001	100
*Species richness*	2.38(0.15)	y = 2.12+0.01x	0.06	0.089	93	4.73(0.11)	y = 6.33−0.05x	0.33	<0.001	100
Cover dominant species	30.08(3.15)			93		31.07(1.26)				100

Some of the variables were logarithmically (log) or square root (sqrt) transformed.

*Bouteloua* and *Larrea* also had opposite effects on the structure and richness of subdominant communities (Appendices 1, 2). The cover of virtually all subdominants grouped according to life forms and life histories decreased as cover of *Bouteloua* increased, whereas cover of subdominants increased as cover of *Larrea* increased. This was true for species of subdominants found only in the *Bouteloua* or *Larrea* communities as well as for those species that occurred in both community types (Appendix S1). Consistent with these trends, species richness of the subdominant communities was negatively related to cover of *Bouteloua*, and positively related to cover of *Larrea*, although this relationship was marginally non-significant for the latter (Appendix S2).

Despite the negative relationship between *Bouteloua* cover and cover of subdominants, after controlling for cover of the dominant species at each vegetation type, cover of forbs, shrubs, summer annuals, and shared species of subdominants was higher in *Bouteloua*- compared to *Larrea*-dominated sites (Appendix S1). In contrast, the cover of winter annuals, perennials, and species unique to each community type did not differ between *Bouteloua*- and *Larrea*-dominated areas. Overall, cover of *Bouteloua* in grassland vegetation was not significantly different than cover of *Larrea* in shrubland areas (ANOVA, *F*_1,191_ = 0.091, *P* = 0.863), but cover of subdominants was slightly higher in *Bouteloua* than in *Larrea* dominated vegetation (ANOVA, *F*_1,190_ = 3.78, *P* = 0.053; Table 2). Thus, despite the apparent facilitation of subdominant species by *Larrea*, total cover of subdominants was lower in *Larrea*-dominated areas (Table 2).

It is conceivable that differences in compositional stability and species richness between grass- and shrub-dominated areas are a function of different environmental conditions [1]. Our study sites at the Sevilleta are only a few kilometers apart so they experience similar seasonal and annual climate variability. Soil conditions (carbon and nitrogen) and annual net primary productivity do not differ between our *Larrea* and *Bouteloua*-dominated sites [44], [49].

**Figure 2:**
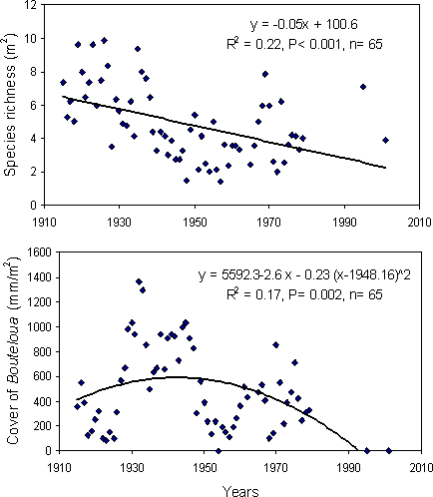
Change in species richness (upper panel) and *Bouteloua* cover (lower panel) from 1915 to 2001 in permanent vegetation quadrats located in *Bouteloua*-dominated areas that were invaded by *Larrea* at the Jornada Basin LTER, New Mexico, USA.

In addition, data from the long-term permanent quadrats at JER show that *Larrea* invaded species-rich *Bouteloua*-dominated sites, and that as *Larrea* invasion progressed through time, total species diversity (Linear regression: *F*_1,65_ = 17.83, R^2^ = 0.22, *P*<0.001) and the cover of *Bouteloua* decreased (Quadratic regression: *F*_1,65_ = 6.43, R^2^ = 0.17, *P* = 0.002; Linear regression: y = 8336.5−4.0x, R^2^ = 0.06, *P* = 0.001, Figure 2). For these analyses, time was used as a proxy for increasing cover of *Larrea*, as woody species were not sampled in the permanent vegetation quadrats but the areas in which these quadrats were located were invaded by *Larrea* during the sampling period. Therefore, this evidence supports the idea that at the SNWR, *Larrea* invasion, and not initial differences in species diversity or environmental factors, was the main cause of species loss in areas that were formerly dominated by C_4_ grasses.

## Discussion

Overall, the dominant species, *Bouteloua* and *Larrea*, had radically different effects on the compositional stability of subdominant communities at the Sevilleta. Although relationships are relatively weak in some cases, the key is how they differ significantly in direction. In particular, compositional stability increased as cover of *Bouteloua* increased and decreased as cover of *Larrea* increased. Our results also suggested that the dominant species had opposite interspecific interactions with subdominant species. That is, total cover of subdominants decreased as cover of *Bouteloua* increased suggesting that *Bouteloua* competes with virtually all groups of subdominants regardless of their life form, life history, and distribution. In contrast, cover of subdominants increased as *Larrea* cover increased, suggesting that *Larrea* may facilitate subdominant species in this aridland ecosystem.

It has been proposed that invasive species disrupt the community properties that maintain species diversity at small spatial scales [50], [51]. However, field studies exploring the mechanisms that cause biodiversity loss during biological invasions are scarce. Our long-term study shows that the invasion of *Larrea* and the local extinction of *Bouteloua* affected community compositional stability, and species diversity. We found that subdominant communities in areas invaded by *Larrea* over 50 years ago at the SNWR differed in their current patterns of community stability, as *Larrea* invaded areas had lower compositional stability compared to communities in *Bouteloua*-dominated vegetation. Thus, our findings confirm predictions from theoretical studies that species invasion can lead to changes in community structure, species interactions, and loss of species diversity. In addition, our study expands on previous studies by demonstrating that species invasion can alter compositional stability by increasing the rate of species turnover.

Recent analyses indicate that species diversity is rarely a primary driver of community stability, when stability is defined as changes in species richness [1], [14]. Although communities with high species diversity may be more resistant to invasion and have higher compensation potential following local extinction events, species invasion and extinction are more likely to cause larger species losses in species rich than in species poor communities [1], [10], [18]. In contrast, a growing body of evidence suggests that the structure of species interactions is a critical determinant of stability in natural communities [3], [16]. Thus, in our aridland ecosystem, lower community stability is likely due to changes in the structure of species interactions due to the replacement of a competitive grass with a predominantly facilitative shrub, rather than to initial differences in species diversity in invaded vs. non-invaded areas, and to species loss after *Larrea* invasion.

Our long-term field observations demonstrated that the compositional stability of subdominant communities depended on the interactions exerted by the dominant species. Thus our results confirm predictions from theoretical and empirical research suggesting that community stability is often determined by one or a few species that have strong effects on the structure of interspecific interactions within a community [12], [52], [53]. Indeed, prior research in grasslands demonstrates that patterns of species dominance may be better predictors of compositional stability than total cover [5]. Furthermore, theoretical work indicates that in competitive communities (e.g., primary producers) the structure of interspecific interactions is the most relevant factor determining community stability [12]. In particular, community models reveal that the mean and variance of interspecific interactions are decisive parameters that dictate community stability [7]. Specifically, these models show that competitive communities are destabilized when an invading species has a large effect on the mean and variance of interspecific interactions within a community [14], [17]. For instance, in communities where weak interactions predominate, the invasion of a strongly interacting species causes large deviations in the mean and variance of overall interspecific interactions and leads to species loss. Although we do not evaluate interspecific interactions in *Bouteloua*- and *Larrea*-dominated communities in detail, other work at SNWR has shown that *Bouteloua* competes with subordinate species whereas *Larrea* facilitates subordinate species [19]. Thus, our empirical results are consistent with theoretical models that show that lower community stability resulted from altered interspecific interactions as a result of the replacement of a competitive by a facilitative species.

As consequence, we argue that in this aridland ecosystem, *Larrea* invasion destabilized subdominant communities because *Larrea* altered the structure of interspecific interactions. That is, the mean and variance of the intensity of interspecific interactions of invaded communities changed because *Larrea* facilitates subdominants instead of competing with them as does *Bouteloua* [19] (Table 2, Appendix S2). These interactions are themselves unstable (or variable), however, because facilitation by shrubs has been shown to have both positive and negative effects on subdominant communities depending on environmental conditions [24], [54]. During periods of extreme heat and drought shrubs facilitate subdominants through water uplift and shade, whereas they compete with subdominants under more favorable environmental conditions [20], [21], [55]. In contrast, *Bouteloua* consistently competes with subdominant species [19], [38], [56]. Hence, the broad range of interactions exerted by *Larrea* on subdominant species may cause drastic changes in the structure of interspecific interactions within shrub-dominated areas which can decrease compositional and community stability [23], while the steady competitive effects of *Bouteloua* on subdominants is likely to reduce species turnover rates and enhance compositional stability. These divergent interactions are further enhanced at our study site because nutrient concentrations and soil moisture although comparable beneath grass and shrub canopies have higher temporal variation beneath the canopy of *Larrea* compared to *Bouteloua* [44], indicating that subdominants beneath *Larrea* are subjected to higher temporal heterogeneity of resources.

Processes responsible for community stability can be linked to the temporal variation (i.e., stability) of different parameters at both the community and population levels. Therefore, examining population and community variation can yield insights into the processes responsible for compositional stability in natural communities. In particular, field and theoretical studies indicate that in plant communities, high species diversity stabilizes community productivity or cover due to population level instability [2], [7], [13]. In our study, however, population and community parameters for compositional stability were counter to those predicted from these studies. In fact, *Larrea* and *Bouteloua* had opposite effects on compositional and population stability, but equally negative effects on the cover stability of subdominants as a whole (Figure 1, Table 1). For instance, as cover of *Bouteloua* increased, subdominants experienced increasing variability in total community cover and population size, while the composition of subordinate species remained stable through time. In contrast, as cover of *Larrea* increased, total community cover and species composition of subdominants were less stable, while population stability was unaffected by cover of *Larrea*. In our system, only the rates of species turnover (colonization and extinction) explained the overall patterns of compositional stability of subdominant communities (Table 1, Figure 1).

It is possible that ten years is not long enough to capture ecologically meaningful links among species invasion/extinction, compositional stability, and patterns of variation of population size in this system. It is also possible that increased rates of species turnover caused population instabilities that led to species extinctions some time in the past. In such case, the present patterns of compositional stability would be relicts of past community dynamics, and do not currently influence other aspects of community stability. Due to the lack of information on community and population dynamics during the time when *Larrea* invasion occurred at SNWR, our results add to the continuing conundrum regarding how population variability contributes to different aspects of community stability [57]. However, these findings support the idea that species interactions and composition play a critical role in defining the stability-diversity relationship [12] and the role of biotic interactions in plant invasions [58]. Therefore, interspecific interactions imposed by dominant species may play a more critical role in defining community stability than previously realized [5].

Although long-term observational data necessarily limits the identification of causes and effects due to the correlative nature of many processes in nature, such data help to identify factors that may be drivers of key ecological processes. In our study, we can not reject the hypothesis that changes in compositional stability result from loss of species diversity at the SNWR. However, diversity per se appears to be a secondary driver of community stability in this system and diversity is itself subject to variations in community stability and environmental factors [1]. In conclusion, our long-term analysis of population and community dynamics demonstrated that *Larrea* invasion into *Bouteloua*-dominated grassland reduced cover of the dominant C_4_ grass and total species richness, as well as total cover and community stability of subordinate species. These changes, in concert with other factors, such as changes in plant chemical compounds [37], altered niche differentiation at the plant scale [19], [23], [59] and decreased niche dimensionality at the community scale due to shifts in soil resource availability [60], may contribute to biodiversity loss as native C_3_ shrubs invade areas formerly dominated by long-lived perennial C_4_ grasses [43]. To the extent that cover reflects abundance, lower cover leads to lower biodiversity by increasing the probability of local extinction through both stochastic (drift) and deterministic (competition) mechanisms [2], [61]. As global change continues to create conditions that favor C_3_ shrubs over C_4_ grasses in arid and semiarid ecosystems worldwide [42], compositional stability will likely decline further through altered species interactions [62], increased environmental variability, and altered disturbance cycles. Therefore, loss of compositional stability may be an increasingly important cause of species loss during biological invasions. In addition, long-term studies of the consequences of invasion by native species can serve as valuable surrogates to understand the impacts of invasion by non-indigenous species on community structure and function under future climate scenarios.

## Supporting Information

Appendix S1 ANCOVA results for the effects of vegetation type (site) and cover of Larrea and Bouteloua on the cover of functional groups, and species richness of subdominant plant communities.
(0.09 MB DOC)

Appendix S2 Linear regressions (solid lines) and means (dotted lines) of the 10-year mean cover and species richness of subdominants as a function of the cover of Larrea and Bouteloua at the Sevilleta NWR.
(0.07 MB TIF)
