# Supplementary material for: Shrub Invasion Decreases Diversity and Alters Community Stability in Northern Chihuahuan Desert Plant Communities
Source: PLoS One. 2008 Jun 4;3(6):e2332. doi: 10.1371/journal.pone.0002332 (PMC2409219; doi:10.1371/journal.pone.0002332)
Supplement: Appendix S1 — ANCOVA results for the effects of vegetation type (site) and cover of Larrea and Bouteloua on the cover of functional groups, and species richness of subdominant plant communities. (0.09 MB DOC) [file pone.0002332.s001.doc]

Appendix 1. ANCOVA’s results for the effects of vegetation type (site) and cover of *Larrea* and *Bouteloua* on the cover of functional groups, and species richness of subdominant plant communities.

| **Factors** | **Estimates** |  |  |  |  |  | **Whole model** | |
| --- | --- | --- | --- | --- | --- | --- | --- | --- |
| ***Life form*** | **Term** | **d.f.** | **Estimate** | **SE** | **t-ratio** | ***P*** | **r^2^** | ***P*** |
| **Forbs (sqrt)** | Intercept |  | 1.342 | 0.071 | 18.86 | <0.001 | 0.12 | <0.001 |
|  | site | 1 | -0.036 | 0.035 | -1.03 | 0.301 |  |  |
|  | cover | 1 | -0.001 | 0.001 | -0.85 | 0.392 |  |  |
|  | cover x site | 1 | 0.007 | 0.001 | 3.82 | <0.001 |  |  |
| **Grasses (sqrt)** | Intercept |  | 1.817 | 0.137 | 13.17 | <0.001 | 0.01 | 0.611 |
|  | site | 1 | 0.059 | 0.069 | 0.86 | 0.387 |  |  |
|  | cover | 1 | -0.004 | 0.003 | -1.15 | 0.25 |  |  |
|  | cover x site | 1 | 0.002 | 0.003 | 0.76 | 0.446 |  |  |
| **Shrubs (sqrt)** | Intercept |  | 1.45 | 0.103 | 14.07 | <0.001 | 0.15 | <0.001 |
|  | site | 1 | 0.18 | 0.051 | 3.49 | <0.001 |  |  |
|  | cover | 1 | -0.004 | 0.002 | -1.7 | 0.091 |  |  |
|  | cover x site | 1 | 0.01 | 0.002 | 3.57 | <0.001 |  |  |
| ***Life history*** |  |  |  |  |  |  |  |  |
| **Summer annuals (sqrt)** | Intercept |  | 1.402 | 0.085 | 16.34 | <0.001 | 0.11 | <0.001 |
|  | site | 1 | -0.152 | 0.042 | -3.54 | <0.001 |  |  |
|  | cover | 1 | -0.001 | 0.002 | -0.47 | 0.636 |  |  |
|  | cover x site | 1 | 0.004 | 0.002 | 2.02 | 0.044 |  |  |
| **Winter annuals (sqrt)** | Intercept |  | 1.009 | 0.074 | 13.58 | <0.001 | 0.21 | <0.001 |
|  | site | 1 | 0.062 | 0.037 | 1.69 | 0.093 |  |  |
|  | cover | 1 | 0.002 | 0.002 | 1.1 | 0.272 |  |  |
|  | cover x site | 1 | 0.007 | 0.002 | 3.79 | <0.001 |  |  |
| **Perennials (sqrt)** | Intercept |  | 1.797 | 0.104 | 17.16 | <0.001 | 0.1 | 0.001 |
|  | site | 1 | 0.161 | 0.052 | 3.07 | 0.002 |  |  |
|  | cover | 1 | -0.006 | 0.002 | -2.35 | 0.02 |  |  |
|  | cover x site | 1 | 0.007 | 0.002 | 2.64 | 0.009 |  |  |
| ***Distribution*** |  |  |  |  |  |  |  |  |
| **Shared (log)** | Intercept |  | 0.569 | 0.029 | 19.52 | <0.001 | 0.08 | 0.007 |
|  | site | 1 | 0.015 | 0.014 | 1.06 | 0.29 |  |  |
|  | cover | 1 | -0.001 | 0.001 | -2.46 | 0.015 |  |  |
|  | site x cover | 1 | 0.002 | 0.001 | 3.4 | <0.001 |  |  |
| **Restricted (log)** | Intercept |  | 0.163 | 0.051 | 3.18 | 0.001 | 0.14 | <0.01 |
|  | site | 1 | 0.032 | 0.025 | 1.25 | 0.21 |  |  |
|  | cover | 1 | 0.001 | 0.001 | 1.01 | 0.309 |  |  |
|  | site x cover | 1 | 0.004 | 0.001 | 2.82 | 0.005 |  |  |
| **Species richness** | Intercept |  | 4.222 | 0.186 | 22.68 | <.0001 | 0.53 | <0.001 |
|  | site | 1 | -1.183 | 0.088 | -13.42 | <.0001 |  |  |
|  | cover | 1 | -0.021 | 0.005 | -4.03 | <.0001 |  |  |
|  | site x cover | 1 | 0.03 | 0.005 | 5.67 | <0.001 |  |  |
