# Supplementary figures and images for: Shrub Invasion Decreases Diversity and Alters Community Stability in Northern Chihuahuan Desert Plant Communities
Source: PLoS One. 2008 Jun 4;3(6):e2332. doi: 10.1371/journal.pone.0002332 (PMC2409219; doi:10.1371/journal.pone.0002332)

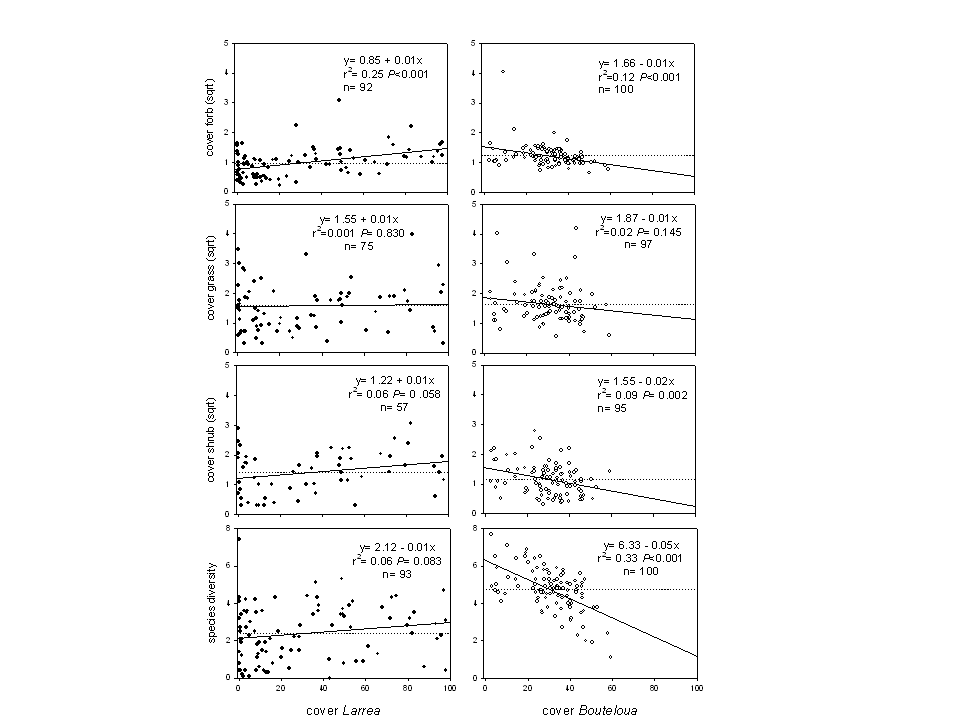

Supplement: Appendix S2 — Linear regressions (solid lines) and means (dotted lines) of the 10-year mean cover and species richness of subdominants as a function of the cover of Larrea and Bouteloua at the Sevilleta NWR. (0.07 MB TIF) [file pone.0002332.s002.tif]
